# Quercetin and polycystic ovary syndrome, current evidence and future directions: a systematic review

**DOI:** 10.1186/s13048-020-0616-z

**Published:** 2020-01-31

**Authors:** Fatemeh Pourteymour Fard Tabrizi, Fatemeh Hajizadeh-Sharafabad, Maryam Vaezi, Hamed Jafari-Vayghan, Mohammad Alizadeh, Vahid Maleki

**Affiliations:** 1 Student Research Committee, Faculty of Nutrition and Food Sciences, Tabriz University of Medical Sciences, Tabriz, Iran; 2 Women’s Reproductive Health Research Center, Tabriz University of Medical Sciences, Tabriz, Iran; 3 Department of Obstetrics and Gynecology, AL Zahra Teaching Hospital, Tabriz University of Medical Sciences, Tabriz, Iran; 4 Faculty of Health, Arak University of Medical Sciences, Arak, Iran; 5 Nutrition Research Center, Faculty of Nutrition and Food Sciences, Tabriz University of Medical Sciences, Tabriz, Iran; 6 Department of Clinical Nutrition, Faculty of Nutrition and Food Science, Tabriz University of Medical Sciences, Tabriz, Iran

**Keywords:** Polycystic ovary syndrome, Quercetin, Insulin resistance, Inflammation, Oxidative stress

## Abstract

Polycystic ovary syndrome (PCOS) is a polygenic endocrine disorder and the most common gynecological endocrinopathy among reproductive-aged women. Current remedies are often used only to control its signs and symptoms, while they are not thoroughly able to prevent complications. Quercetin is an herbal bioactive flavonoid commonly used for the treatment of metabolic and inflammatory disorders. Thus, this systematic review was conducted to evaluate the efficacy of quercetin supplementation in subjects with PCOS. Databases until March 2019 were searched. All human clinical trials and animal models evaluating the effects of quercetin on PCOS women were included. Out of 253 articles identified in our search, 8 eligible articles (5 animal studies and 3 clinical trials) were reviewed. The majority of studies supported the beneficial effects of quercetin on the ovarian histomorphology, folliculogenesis, and luteinisation processes. The effects of quercetin on reducing the levels of testosterone, luteinizing hormone (LH), and insulin resistance were also reported. Although quercetin improved dyslipidemia, no significant effect was reported for weight loss. It is suggested that the benefits of quercetin may be more closely related to antioxidant and anti-inflammatory features of quercetin rather than weight-reducing effects. Therefore, this review article provides evidence that quercetin could be considered as a potential agent to attenuate PCOS complications. However, due to the paucity of high-quality clinical trials, further studies are needed.

## Introduction

Polycystic ovary syndrome (PCOS), as a polygenic endocrine disorder, is the most common gynecological endocrinopathy, which is estimated to affect 2–20% of reproductive-aged women [1]. Common physiological manifestations of this syndrome include ovarian enlargement, hyperandrogenism, androgenic alopecia, hirsutism, acne, menstrual irregularity, anovulation or oligo-amenorrhea, miscarriage, and infertility [2]. PCOS-related symptoms also impair the quality of life through affecting psychiatric aspects of patient’s life [3]. Women with PCOS exhibit an increased incidence of several chronic diseases including obesity, dyslipidemia, hypertension, heart disease, and type 2 diabetes mellitus (T2DM) [4–6]. It is documented that both obese and lean women with PCOS mostly exhibit insulin resistance, a major risk factor for the development of metabolic abnormalities such as impaired glucose tolerance (IGT) and T2DM [7, 8]. According to recent studies, oxidative stress is demonstrated as another contributing factor in the development of PCOS and its corresponding symptoms such as increased androgen production and infertility [9]. Although the definite etiology of PCOS remains unclear, complex interactions between genetic, behavioral, and environmental factors play critical roles in the development of PCOS and subsequent therapeutic options [10]. Current remedies are often used only to control its signs and symptoms, while they are not thoroughly able to prevent complications. Therefore, natural products became a topic of interest for the management of PCOS and its complications [11]. Herbal plants are extensively used to prevent chronic diseases due to their polyphenolic compounds, multi-targeted effectiveness, and low toxicity [12]. Quercetin is an herbal bioactive flavonoid with radical scavenging and antioxidant properties, which is extensively used for the treatment of metabolic and inflammatory disorders [13]. Fruits and vegetables, particularly onions, apples, berries, citrus, red grapes, nuts, seeds, and tea, are a good source of quercetin [14]. Several pharmacological studies revealed that, Quercetin supplements are effective in the regulation of redux status [15], reducing inflammation [16], protecting cardiovascular system [17], inhibiting platelet aggregation [18], relaxing vessels smooth muscles [19], and preventing LDL oxidation [20], hypertension [21], cancer development [22, 23] and diabetes [24]. It is postulated that hypoglycemic effect of the quercetin is related to insulin signal transduction, such as enhanced protein expression, and tyrosine phosphorylation of insulin receptor (IR), several insulin receptor substrates (IRSs) and glucose transporters (GLUTs) [25]. Moreover, quercetin exerts the insulin-sensitizing effect by promoting the proliferation of pancreatic β-cells and enhancing glucose metabolism and insulin secretion [26]. Oxidative stress is considered as a potential stimulant of PCOS, and serum levels of antioxidants are reduced in patients with PCOS. Therefore, the use of anti-oxidative agents in the management of PCOS has attracted a lot of attention [27, 28]. Quercetin could be considered as an anti-oxidative agent due to its ability to inhibit xanthine oxidase through reducing the generation of free radicals, modifying antioxidants and inhibiting lipid peroxidation [29].

The current study aimed to systematically summarize the scientific literature regarding therapeutic effects of quercetin on PCOS, as the most common endocrinopathy in reproductive-aged women.

## Methods

### Search strategy

Literature search was conducted in PubMed, Scopus, Embase, ProQuest and Google Scholar electronic databases using the keywords “quercetin” [Title/Abstract] AND “polycystic ovary syndrome” [Title/Abstract] OR “PCOS” [Title/Abstract] OR “sclerocystic ovary syndrome” [Title/Abstract] OR “dysmetabolic Syndrome” [Title/Abstract]. Reference lists and related records were also reviewed. The search was limited to English language papers published until March 2019. Guideline of the Preferred Reporting for Systematic Reviews (PRISMA) was used for designing this systematic review.

### Eligibility criteria

We included all human clinical trials, as well as animal studies, and published in English-language journals. In-vitro models, reviews studies, non-English-language articles and those with no access to the full text were excluded.

### Data extraction

The titles and abstracts of the eligible papers were independently screened by two researchers. Studies were excluded if they could not meet the criteria. To extract data, eligible articles were evaluated based the on an aim checklist, research question, and inclusion/exclusion criteria. Then, the quality of the included studies was assessed by a third reviewer. In case of any disagreements, a third author was consulted.

## Results

### Selected articles

**Fig. 1:**
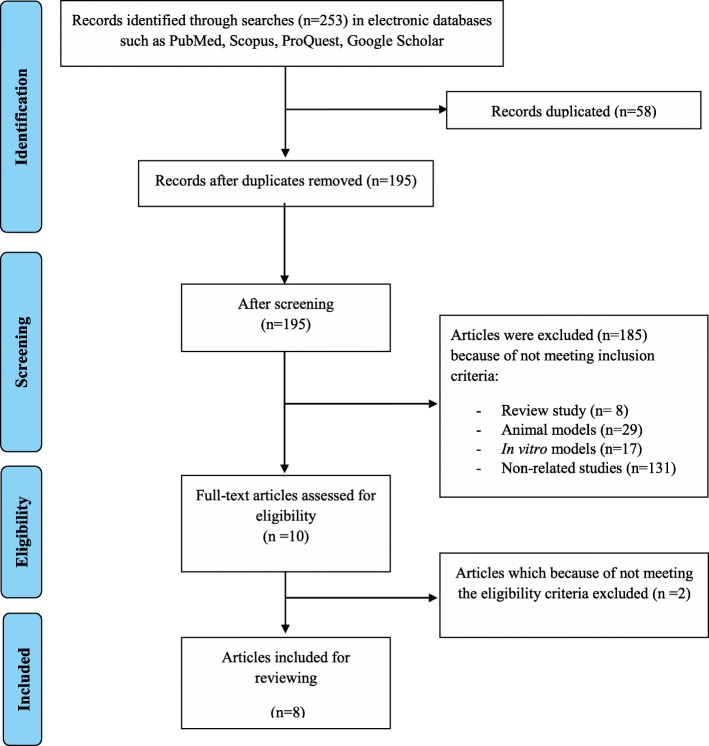
Flow diagram of the literature search and study selection process

Figure 1 presents a flow diagram of the study selection. Totally, 253 articles were retrieved, 58 of which were duplicates resulting in 195 non-duplicated publications. One hundred eighty five articles could not meet our inclusion criteria and were excluded. Also, two articles were excluded due to not meeting the eligibility criteria. Finally, only 8 full-text articles were consistent with the purpose of this study and were reviewed (Table 1).

**Table 1:** Characteristics of studies that reported the roles of quercetin in polycystic ovary syndrome

Type of study	Authors/date	Source	Model	Results
Animal	Wang et al. 2017 [30]	China	132 female Wistar rats (21 days old)/IR PCOS rat model/2 mL of quercetin solution (100 mg/kg)/ for 28 days	Quercetin treatment in the insulin-resistant PCOS rat model led to:
				- 58.33% recovery rate of the estrous cycle, significant reduced the levels of blood insulin, interleukin 1β, IL-6, and tumor necrosis factor α.
				- Significant decreased the granulosa cell nuclear translocation of NF-κB
				- Inhibited the expression of inflammation-related genes, including the nicotinamide adenine dinucleotide phosphate oxidase subunit p22phox, oxidized low-density lipoprotein, and Toll-like receptor 4, in ovarian tissue.
				- IR improvement
	Jahan et al. 2018 [31]	Pakistan	Twenty-four adult female Sprague Dawley rats (60–70 days old and 180 ± 10 g body weight; randomly divided into four groups (*n* = 6–8))/quercetin (30 mg/kg) for 21 days.	By Quercetin administration:
				- No difference in mean body weight
				-Restoration of the estrous cycle
				-Significant decrease in ovarian diameter and in cystic follicle diameter
				-Number of ovarian follicles were declined as compared to untreated PCOS group
				-Counterbalanced the ROS levels and improved the antioxidant activities
				- Optimized the values of progesterone, estradiol, and testosterone levels when compared to control
				-Improvement of lipid profile (decreased cholesterol and triglyceride levels) and glucose levels.
	Neisy et al. 2018 [32]	Iran	Thirty-five Sprague–Dawley female rats (DHEA-induced PCOS) randomly divided into five groups: (1) Control group, didn’t receive any treatment for 30 days; (2) quercetin (Q) group, treated with quercetin gavage (15 mg kg^−1^quercetin (3) ethanol vehicle group (ethanol gavage) for 30 days; (4) PCOS group (5) PCOSQ group (induced PCOS and then were treated with 15 mg kg^− 1^ quercetin for 30 days). 15 mg kg^_1^ quercetin for 30 days	Quercetin significantly:
				-Improved folliculogenesis and luteinisation
				-Improved IR and decreased insulin levels
				-Increased activities of liver GK and HK
				↑ Expression of uterine GLUT4 and ERa genes
	Shah et al. 2016 [33]	India	Forty-eight Sprague–Dawley female rats (3-week-old)/Quercetin (150 mg/kg, p.o.)/4 week.	Quercetin led to:
				- ↓ CYP17A1 gene expression
				- PI3 kinase inhibition
				-Decreased testosterone and LH levels
				-Significant improvement in insulin, testosterone, LH, and lipid profile (decreased HDL level was improved and significant reduction in serum cholesterol, triglyceride, LDL, and VLDL levels)
				-Significant improvement in the uterus histology
				-Improvement in cyst formation, folliculogenesis, and luteinisation
				- Did not modify body weight gain
	Hong et al. 2018 [34]	China	Sprague–Dawley female rats. (25 mg Quercetin /kg body weight for 4 week.	Quercetin led to:
				-Reversed the PCOS ovarian morphology.
				**-↑** The levels and activities of antioxidant enzymes: CAT, SOD and GPX
				-Prevented weight gain
				-Caused significant decline in serum glucose
				-Normalized estradiol, testosterone levels, and steroidogenic enzyme activities in PCOS subjects
				-Blocked PCOS-related abnormalities and exerted protective effects on the ovary anatomy.
Human	Rezvan et al. 2017 [35]	Iran	84 women with PCOS (20–40 years old; and had the BMI of 25–40 kg/m^2^) randomly assigned to 2 groups. The treatment group received 1 g quercetin (two 500 mg capsules (Jarrow, USA) after each main meal (breakfast and lunch) for 12 weeks. The control group received placebo(2 capsules containing starch for 12 weeks)	Quercetin led to:
				-Increased the level of adiponectin by 5.56% and HMW adiponectin by 3.9% reduced the level of testosterone, LH, and HOMA-IR levels were also significantly reduced in quercetin group reduced of FBS, and insulin levels without changing BMI and WHR
				-Oral quercetin supplementation was effective in improving the adiponectin-mediated insulin resistance and hormonal profile of women with PCOS.
	Khorshidi et al. 2018 [36]	Iran	78 overweight or obese women (25 ≤ BMI ≤ 40 kg/m2, 20–40 years) with PCOS 1000 mg/day quercetin or placebo for 12 weeks	Quercetin led to:
				Decreased resistin plasma levels and gene expression, and testosterone and LH concentration
				No significant difference in SHBG levels
				FBG, fasting insulin, and insulin resistance were improved significantly in the quercetin group, but the changes were not statistically different compared with the placebo group
	Rezvan et al. 2018 [37]	Iran	84 overweight or obese women with PCOS/1 g quercetin (two 500 mg capsules) daily for 12 weeks	Quercetin:
				Increased Adiponectin Receptors *(ADIPOR1* and *ADIPOR2)* transcript expression by 1.32- and 1.46-fold respectively,
				Enhanced AMPK level by 12.3%

Abbreviations: *ADIPORs* Adiponectin Receptors, *AMPK* AMP-activated protein kinase, *BMI* Body mass index, *CYP17A1* Cytochrome P450 17A1, *CAT* Catalase, *DHEA* Dehydroepiandrosterone, *Erα* Oestrogen receptor α, *FBG* Fasting blood glucose, *GK* Glucokinase, *GLUT4* Glucose transporter 4, *GPX* Glutathione peroxidase, *HDL* High-density lipoprotein, *HK* Hexokinase, *HMW* High molecular weight, *HOMA-IR* Homeostasis model of assessment-insulin resistance, *IL-6* Interleukin 6, *IR* Insulin resistance, *LDL* Low-density lipoprotein, *LH* Luteinizing hormone, *NF-κB* Nuclear factor kappa-light-chain-enhancer of activated B cells, *PCOS* Polycystic ovary syndrome, *PI3K* Phosphatidyl inositol 3-kinase, *ROS* Reactive oxygen species, *SHBG* Sex hormone binding globulin, *SOD* Super oxide dismutase, *TBARS* Thiobarbituric acid reactive substances, *VLDL* Very low-density lipoprotein

### Quercetin and weight changes in PCOS

In these studies, the presence of PCOS is a contributing factor in the increased incidence of weight gain and overweight. Two of 5 animal studies reported that administration of Quercetin prevented weight gain and caused significant decrease in body weight in PCOS rats [30, 34]. Other animal studies [31–33] as well as 3 human trials [35–37] showed insignificant changes in weight, waist circumference and BMI among intervention groups compared to control groups.

### Quercetin and ovarian histomorphology in PCOS

Five of eight studies used histological analysis including ovarian morphology, weight and diameter, and reported a significant improvement in ovarian morphology, folliculogenesis, and luteinisation after treatment with quercetin [30–34, 37]. Jahan et al. [31] reported a significant decrease in ovarian diameter and cystic follicle diameter in quercetin-treated PCOS subjects. Also, treatment with quercetin normalized the thickness of theca and granulosa layer. Overall, these studies found that treatment with quercetin increase normal follicles in ovaries, restore the anatomy of normal ovary, and improve the histology of the uterus, which are comparable to metformin [31–34].

### Quercetin and reproductive hormones in PCOS

Six of eight studies examined the effects of quercetin on the reproductive hormones in PCOS subjects [31, 33–37]. It is reported that administration of quercetin improved the estrous cyclicity of the PCOS subjects [30, 31]. Hong Y et al. [34] showed that quercetin at a dose of 25 mg/kg body weight decreased the activity of steroidogenic enzymes (3β-HSD and/or 17β-HSD), in rat model with PCOS. Moreover, quercetin could regulate steroidogenesis through reducing testosterone levels and improving progesterone and estradiol levels [31]. The reducing effects of quercetin administration on testosterone, LH, and estradiol levels in PCOS subjects were also reported by five other studies [31, 33–36]. Findings from two human studies showed that quercetin supplementation in women with PCOS slightly improved testosterone and LH level, but the effects on sex hormone binding globulin (SHBG) was marginal [35, 36]. Hirsutism, a condition of unwanted hair growth in PCOS due to excess androgens, was evaluated by two of eight studies [31, 33]; however, all the included studies confirmed that quercetin treatment could successfully improve subsiding hirsutism. Two studies showed that administration of quercetin led to decreased expression of CYP17A1 gene which is responsible for the activity of 17a-hydroxylase, a key enzyme for androgen synthesis [31, 33].

### Quercetin and metabolic profile in PCOS

Seven of eight studies evaluated the potential effects of quercetin on the metabolic profile in PCOS subjects [30, 32–37]. Hong et al. [34] reported that quercetin intake at a dose of 25 mg/kg resulted in a reduction of plasma glucose levels in PCOS rats. Other studies assessed the changes in the insulin and glucose levels, as well as homeostasis model assessment of insulin resistance (HOMA-IR) values following quercetin intake [30, 32, 33, 35, 37]. They reported that quercetin intake caused significant decrease in these values and improved insulin resistance in PCOS cases. Daily consumption of 1000 mg quercetin for 12 weeks reduced insulin resistance and fasting levels of insulin and glucose as well as plasma concentration and gene expression of resistin in overweight or obese women with PCOS; however, these changes were not statistically significant after controlling the potential confounders [36]. It is shown that adiponectin regulates the reproductive system by blocking the secretion of LH and GnRH [38]. Rezvan et al. [35] showed that oral quercetin supplementation increases the serum levels of total adiponectin by 5.56% and high-molecular-weight (HMW) adiponectin by 3.9% in PCOS women as compared to placebo. They showed the efficient role of quercetin in improving the adiponectin-mediated insulin resistance and hormonal profile of women with PCOS. In another study, quercetin supplementation up-regulated transcript expression levels of adiponectin receptors (ADIPOR1, ADIPOR2) [37]. Furthermore, they reported that quercetin increases the levels of AMP-activated protein kinase (AMPK) by 12.3% compared to the control group. AMPK enhances the regulation of glucose transporter 4 (GLUT4) as a key sensor of energy, and therefore induces glucose uptake. Treatment with quercetin for 30 days significantly increased the activity of liver hexokinase (HK) and glucokinase (GK) to a normal level in the uterus of PCOS subjects compared to the untreated group [34]. Moreover, the treatment increased the gene expression of estrogen receptor alpha (ER α) and GLUT4 up to 5 and 4.4 folds, respectively.

### Quercetin and dyslipidemia in PCOS

Two of 8 studies investigated lipid profile following quercetin supplementation in PCOS subjects. They reported that treatment with quercetin produce a significant reduction in serum levels of TC, TG, LDL-C, and very low density lipoprotein (VLDL) and a significant increase in HDL-C levels compared to the control group [31, 33].

### Quercetin and oxidative stress and inflammation in PCOS

Effectiveness of quercetin on oxidative stress and inflammation in PCOS were assessed in three out of 8 studies [30, 31, 34]. Administration of quercetin inhibited the expression of inflammation-related genes including the nicotinamide adenine dinucleotide phosphate oxidase subunit (p22phox), oxidized low-density lipoprotein (OX-LDL), and Toll-like receptor 4 (TLR-4), in ovarian tissue of PCOS subjects [30]. Quercetin also decreased the messenger RNA (mRNA) and protein levels of p22phox, OX-LDL, and TLR-4. Besides, quercetin significantly reduced the blood levels of interleukin 1β (IL-1β), interleukin-6 (IL-6), and tumor necrosis factor α (TNF-α). Also, the granulosa cell nuclear translocation of NF-κB was significantly reduced following quercetin administration in the insulin-resistant PCOS subjects. Neisy et al. indicated that the decrease in insulin resistance as a result of quercetin intake in the PCOS group might be associated with its anti-inflammatory properties such as inhibitory effects on TNF-α [32]. Total protein content and the levels of catalase (CAT), superoxide dismutase (SOD), peroxidase (POD), and glutathione reductase (GR) in ovarian tissue of PCOS subjects were extremely lower than the control [31]. Quercetin treatment reversed these values near to baseline levels. The levels of thiobarbituric acid reactive substances (TBARS), as surrogate marker of lipid peroxidation, were significantly higher in PCOS women than control. Following quercetin administration, TBARS level was decreased in PCOS subjects. Quercetin also significantly increased the activity of SOD, CAT, and glutathione peroxidase (GPX) in the PCOS group [34].

## Discussion

The current systematic review suggests that quercetin possesses an intrinsic potential to correct hormonal disturbances and subsequent metabolic disorders occurred in PCOS. The ovarian follicles of patients with PCOS are large, with a thickened theca cell layer and a degenerated granulosa cell progressed to form cysts [33, 39]. Evidence suggests that the effects of quercetin on the improvement of ovarian histological and histomorphological analysis are similar to or higher than metformin. Animal models suggest beneficial effects of quercetin on folliculogenesis and luteinisation processes through improved ovarian tissue along with the prominent decrease of atretic and cystic follicles, improvement of hirsutism, marked an increase in normal follicles with different stages and vascularization of the thecal layer [31–33]. More luteal together with an increase in the thickness of ovarian granulosa cells were also reported following quercetin administration; however, the changes in the ovaries weight were not statistically significant [30].

Histological changes of the ovarian follicles seem to be mediated by elevated levels of androgens and disrupted folliculogenesis resulting from an irregular estrous cycle [40]. Quercetin decreased testosterone levels and reversed the low levels of estradiol and progesterone to near-normal levels [31]. Studies also reported the existence of more corpora lutea accounting for the restoration of the estrous cycle [31, 41]. It is in line with the reduced activities of 3β-hydroxysteroid dehydrogenase and 17β-hydroxysteroid dehydrogenase and the modified concentrations of testosterone and estradiol, as well as ovary structure following administration of quercetin [34].

**Fig. 2:**
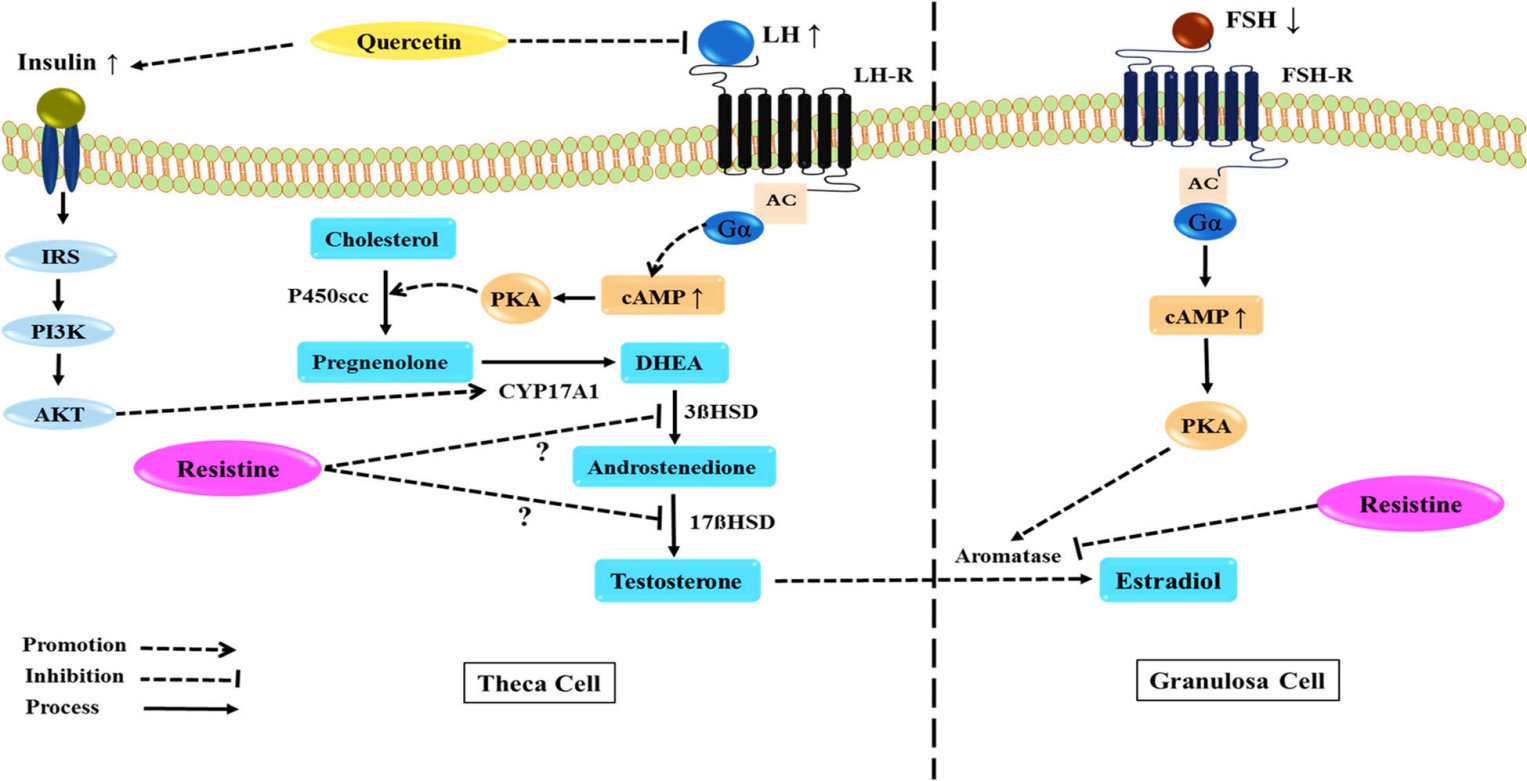
Possible mechanisms of the quercetinpotential roles on hormonal status in ovarian cells. Abbreviations: IRS; Insulin Receptor Substrate, PI3K;Phosphoinositide 3-kinase, AKT; Protein kinase B, P450scc; Cholesterol side-chain cleavage enzyme, CYP17A1; Cytochrome P450 Family 17 Subfamily A Member 1, 3ßHSD; 3β-Hydroxysteroid dehydrogenase, 17ßHSD; 17β-Hydroxysteroid dehydrogenase, DHEA; Dehydroepiandrosterone

Indeed, the evidence suggests that quercetin is able to counteract the mechanisms of androgen biosynthesis related to LH [31, 33]. The increased levels of LH in PCOS, which are due to the impaired hypothalamic-pituitary axis, stimulate PI3K/Akt pathway leading to the over expression of ovarian CYP17A1 gene together with 17-α hydroxylase enzyme levels, which catalyze the conversion of progesterone to androgens [31, 42, 43]. Two studies reported that the lowering effects of quercetin on the levels of testosterone and LH are linked to resistin, as a possible agent in the steroidogenesis [36]. The potential mediators by which resistin elevates steroidogenesis include LH receptor, steroidogenic acute regulatory protein, and insulin receptor [44]. Resistin not only stimulates the synthesis of androgens by increasing theca cells thickness and 17α-hydroxylase activity [45], but also potentiate the activities of 3β-hydroxysteroid dehydrogenase and 17β-hydroxysteroid dehydrogenases accompanied with the increased release of androgens [46]. Resistin, along with LH, also up-regulate gene expression of insulin receptor leading to overproduction of androgens [44]. It was found that quercetin effectively ameliorates serum levels of testosterone and LH through reducing resistin levels [36]. In summary, Fig. 2 indicates the possible mechanisms of the quercetin potential roles on hormonal status in ovarian cells. Hyperandrogenaemia may lead to PCOS-related excessive weight gain and insulin resistance [47]. Moreover, chronic inflammation and oxidative stress, as two hallmarks of PCOS, are closely related in PCOS women and cause impaired insulin action and hyperinsulinemia [48, 49] leading to anovulation [50]. Although quercetin could not make significant changes in body weight in PCOS subjects [31, 33, 34], there is evidence suggesting the ability of quercetin to redistribute fat mass [51]. Quercetin has also exhibited a potential capacity to sensitize insulin receptors attributed to its antioxidant and anti-inflammatory features. The overproduction of ROS derived from NADPH oxidation in PCOS condition leads to higher levels of oxidized LDL (ox-LDL) [52]. The interaction between ox-LDL and Toll-like receptor 4 (TLR4) activates NF-κB path way resulting in elevated expression of pro-inflammatory cytokines including IL-1β, IL-6, and TNF-α which induce insulin resistance [30, 53]. Wang et al. [30] showed that quercetin suppresses the expression of NADPH oxidase subunit p22phox, ox-LDL, and TLR-4 and thereby inhibit TLR-4-NF-κB signaling pathway leading to reduced insulin resistance.

In addition, investigations demonstrated that increasing the levels of antioxidant enzymes such as SOD, CAT, POD, GR, GSHPX, and NADPH oxidase by quercetin, are the other protective effects of quercetin against oxidative stress [31, 34]. Since the oxidative stress is positively related to testosterone levels, insulin resistance, ovarian mesenchyme hyperplasia, and infertility [54, 55]; quercetin may be considered as a potential agent to attenuate PCOS complications. Another possible mechanism by which quercetin reduces insulin resistance is mediated by its beneficial effect on the dominant adipokine regulating insulin resistance named adiponectin [37]. AdipoR1 and AdipoR2 are the prominent receptors of adiponectin which interact with the substrate and activate several signaling cascades such as AMP-activated protein kinase (AMPK) to regulate glucose and fatty acid metabolism [56]. Rezvan et al. [37] indicated that quercetin up-regulates gene expression of *ADIPOR1* and *ADIPOR2* along with a marked increment in AMPK levels in patients with PCOS.

In another study by Khorshidi et al. [36] FBG, insulin, and HOMA-IR were improved after supplementation with quercetin; however, the differences between quercetin and placebo groups were not significant. The authors attributed the glycaemia-mimic effect of quercetin to resistin, which showed a significant decrease in the quercetin group [36]. Resistin diminishes the phosphorylation of AMPK and Akt pathways and decreases the production of IRS-1 as well as tyrosine phosphorylation of IRS-1, leading to increased insulin resistance [57, 58]. Resistin promoter has several binding sites for sterol regulatory element-binding protein 1c and CCAAT enhancer-binding protein alpha (C/EBPα), which bind to resistin promoter and overexpress resistin gene [59].

Quercetin potentially decreases resistin through down-regulating gene expression of C/EBPα [60]. Several indirect mechanisms have been suggested for quercetin to counteract insulin resistance [32]. Lower activity of GK coupled with the increased rate of HK activity observed in PCOS indicates that liver cells in IR state potentiate the activity of HK to compensate decreased GK activity and improve glucose metabolism in the liver [32, 61]. Quercetin increased the activity of both enzymes along with the expression of GLUT4 and reversed lowered activity of GK in PCOSrat liver [32].

**Fig. 3:**
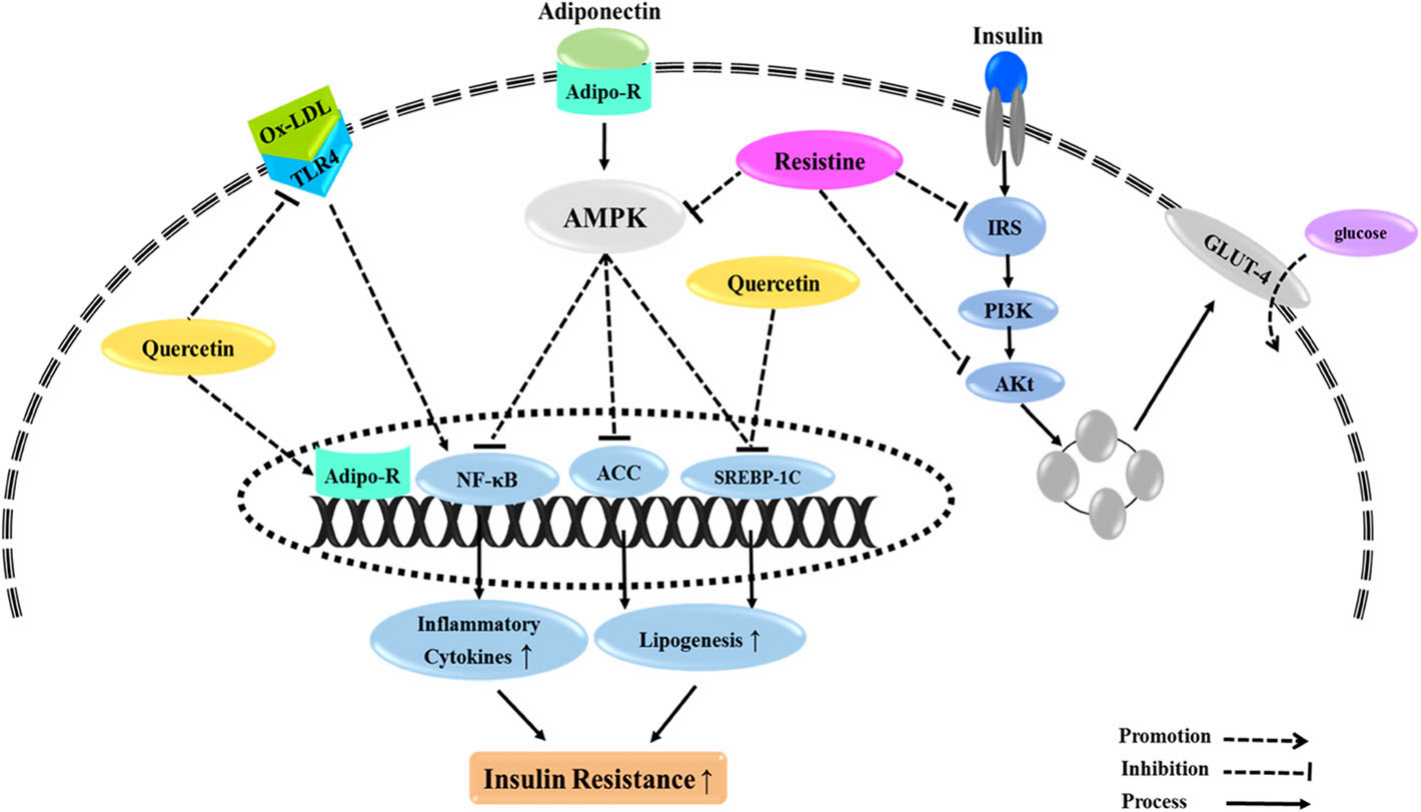
Possible mechanisms of quercetineffects on metabolic indicators. AMPK; AMP-activated protein kinase, IRS;Insulin Receptor Substrate, AKT; Protein kinase B, GLUT4; Glucose transporter type 4, SREBP-1C; Sterol regulatory element-binding protein 1, ACC; Acetyl-CoA carboxylase, NF-κB;nuclear factor kappa-light-chain-enhancer of activated B cells, TLR4; Toll-like receptor 4, Ox-LDL; Oxidized Low-Density Lipoprotein

Dyslipidemia, as a common metabolic disorder in PCOS, appears to occur due to the hormonal imbalance and insulin resistance [62, 63]. Although the available evidence suggests a highly significant improvement in lipid profile following quercetin administration, more studies are required to confirm these beneficial effects. Based on these studies, anti-hyperlipidemic potential of quercetin can be attributed to its capacity in correcting hyperinsulinemia and hyperandrogenemia [31, 33]. In general, the possible mechanisms of quercetin effects on metabolic indicators are summarized in Fig. 3.

### Knowledge gaps and future directions

None of the included studies measured blood concentrations of quercetin to determine its bioavailability. Dose-dependent experiments are also recommended to evaluate the therapeutic effects of quercetin in the management of PCOS complications following each dose, and to determine the optimum dose of quercetin in which the need for metformin is reduced. Since body composition can directly affect hormone levels, glycemic status, and lipid profile, further studies are needed to address the effect of quercetin on body composition. Although insulin resistance is one of the common events in PCOS, only a few studies have examined the impact of quercetin on insulin resistance and the involved mechanisms. Therefore, future well-designed studies with longer duration are warranted to reach conclusive results about quercetin consumption in women with PCOS.

## Conclusion

The current evidence indicates that supplementation with quercetin effectively ameliorate hyperandrogenaemia and irregular estrous and consequently, improve the folliculogenesis and luteinisation processes. However, there are not enough studies to make robust conclusions. While data on the impact of quercetin on metabolic profiles is relatively scarce, there is evidence that quercetin may be able to counteract PCOS complications by improving insulin resistance and chronic inflammation. Mechanisms by which quercetin suppress insulin resistance include reducing testosterone, LH and resistin levels and increasing adiponectin activity.

## Data Availability

All data generated or analyzed during this study are included in this published article.

## Abbreviations

17ßHSD: 17β-Hydroxysteroid dehydrogenase
3ßHSD: 3β-Hydroxysteroid dehydrogenase
ACC: Acetyl-CoA carboxylase
ADIPORs: Adiponectin Receptors
AKT: Protein kinase B
AMPK: AMP-activated protein kinase
BMI: Body mass index
C/EBPα: CCAAT enhancer-binding protein alpha
CAT: Catalase
CYP17A1: Cytochrome P450 17A1
DHEA: Dehydroepiandrosterone
ER α: Estrogen receptor alpha
FBG: Fasting blood glucose
GK: Glucokinase
GLUT4: Glucose transporter 4
GPX: Glutathione peroxidase
GR: Glutathione reductase
HDL: High-density lipoprotein
HDL-C: High density lipoprotein cholesterol
HK: Hexokinase
HMW: High molecular weight
HOMA-IR: Homeostasis model assessment of insulin resistance
IGT: Impaired glucose tolerance
IL-1β: Interleukin 1β
IL-6: Interleukin 6
IR: Insulin resistance
IRS: Insulin Receptor Substrate
LDL-C: Low density lipoprotein cholesterol
LH: Luteinizing Hormone
NF-κB: Nuclear factor kappa-light-chain-enhancer of activated B cells
OX-LDL: Oxidized low-density lipoprotein
P450scc: Cholesterol side-chain cleavage enzyme
PCOS: Polycystic ovary syndrome
PI3K: Phospho inositide 3-kinase
POD: Peroxidase
ROS: Reactive oxygen species
SHBG: Sex hormone binding globulin
SOD: Superoxide dismutase
SREBP-1C: Sterol regulatory element-binding protein 1
T2DM: Type 2 diabetes mellitus
TBARS: Thiobarbituric acid reactive substances
TC: Total cholesterol
TG: Triglyceride
TLR4: Toll-like receptor 4
TNF-α: Tumor necrosis factor α
VLDL: Very low density lipoprotein

## References

[1] Ding T, Hardiman PJ, Petersen I, Wang F-F, Qu F, Baio G (2017). The prevalence of polycystic ovary syndrome in reproductive-aged women of different ethnicity: a systematic review and meta-analysis. Oncotarget..

[2] Sirmans SM, Pate KA (2014). Epidemiology, diagnosis, and management of polycystic ovary syndrome. Clin Epidemiol.

[3] Blay SL, Aguiar JVA, Passos IC (2016). Polycystic ovary syndrome and mental disorders: a systematic review and exploratory meta-analysis. Neuropsychiatr Dis Treat.

[4] Essah PA, Nestler JE (2006). The metabolic syndrome in polycystic ovary syndrome. J Endocrinol Invest.

[5] Beydoun HA, Stadtmauer L, Beydoun MA, Russell H, Zhao Y, Oehninger S (2009). Polycystic ovary syndrome, body mass index and outcomes of assisted reproductive technologies. Reprod Biomed Online.

[6] Orio F, Muscogiuri G, Nese C, Palomba S, Savastano S, Tafuri D (2016). Obesity, type 2 diabetes mellitus and cardiovascular disease risk: an uptodate in the management of polycystic ovary syndrome. Eur J Obstet Gynecol Reprod Biol.

[7] McEwen B, Hartmann G (2018). Insulin resistance’and polycystic ovary syndrome (PCOS)’: part 1. The impact of insulin resistance. J Aust Tradit Soc.

[8] Legro RS, Kunselman AR, Dodson WC, Dunaif A (1999). Prevalence and predictors of risk for type 2 diabetes mellitus and impaired glucose tolerance in polycystic ovary syndrome: a prospective, controlled study in 254 affected women. J Clin Endocrinol Metab.

[9] Shukla A, Mandel L. Polycystic ovarian syndrome. Obes Manag. 2019:1:31–40.

[10] Kakoly NS, Earnest A, Teede HJ, Moran LJ, Joham AE (2019). The impact of obesity on the incidence of type 2 diabetes among women with polycystic ovary syndrome. Diabetes Care.

[11] Rocca ML, Venturella R, Mocciaro R, Di Cello A, Sacchinelli A, Russo V (2015). Polycystic ovary syndrome: chemical pharmacotherapy. Expert Opin Pharmacother.

[12] David AVA, Arulmoli R, Parasuraman S (2016). Overviews of biological importance of quercetin: a bioactive flavonoid. Pharmacogn Rev.

[13] Lakhanpal P, Rai DK (2007). Quercetin: a versatile flavonoid. Internet J Med Updat.

[14] Boots AW, Haenen GRMM, Bast A (2008). Health effects of quercetin: from antioxidant to nutraceutical. Eur J Pharmacol.

[15] Lesjak M, Beara I, Simin N, Pintać D, Majkić T, Bekvalac K (2018). Antioxidant and anti-inflammatory activities of quercetin and its derivatives. J Funct Foods.

[16] Li Y, Yao J, Han C, Yang J, Chaudhry M, Wang S (2016). Quercetin, inflammation and immunity. Nutrients..

[17] Gormaz JG, Quintremil S, Rodrigo R (2015). Cardiovascular disease: a target for the pharmacological effects of quercetin. Curr Top Med Chem.

[18] Faggio C, Sureda A, Morabito S, Sanches-Silva A, Mocan A, Nabavi SF (2017). Flavonoids and platelet aggregation: a brief review. Eur J Pharmacol.

[19] Shen Y, Croft KD, Hodgson JM, Kyle R, Lee I-LE, Wang Y (2012). Quercetin and its metabolites improve vessel function by inducing eNOS activity via phosphorylation of AMPK. Biochem Pharmacol.

[20] Bondonno NP, Bondonno CP, Hodgson JM, Ward NC, Croft KD (2015). The efficacy of quercetin in cardiovascular health. Curr Nutr Rep.

[21] Perez-Vizcaino F, Duarte J, Jimenez R, Santos-Buelga C, Osuna A (2009). Antihypertensive effects of the flavonoid quercetin. Pharmacol Rep.

[22] Baghel SS, Shrivastava N, Baghel RS, Agrawal P, Rajput S (2012). A review of quercetin: antioxidant and anticancer properties. World J Pharm Pharm Sci.

[23] Rauf A, Imran M, Khan IA, ur-Rehman M, Gilani SA, Mehmood Z (2018). Anticancer potential of quercetin: a comprehensive review. Phyther Res.

[24] Shi G-J, Li Y, Cao Q-H, Wu H-X, Tang X-Y, Gao X-H (2019). In vitro and in vivo evidence that quercetin protects against diabetes and its complications: a systematic review of the literature. Biomed Pharmacother.

[25] Eid HM, Haddad PS (2017). The antidiabetic potential of quercetin: underlying mechanisms. Curr Med Chem.

[26] Gurav M, Bhise S, Warghade S (2018). Effect of Quercetin on Beta cell regeneration. Asian J Pharm Pharmacol.

[27] Murri M, Luque-Ramírez M, Insenser M, Ojeda-Ojeda M, Escobar-Morreale HF (2013). Circulating markers of oxidative stress and polycystic ovary syndrome (PCOS): a systematic review and meta-analysis. Hum Reprod Update.

[28] Aquino CI, Nori SL (2014). Complementary therapy in polycystic ovary syndrome. Transl Med UniSa.

[29] Bentz AB. A review of Quercetin: chemistry, Antioxident properties, and bioavailability. J Young Investig. 2017;10:1–15.

[30] Wang Z, Zhai D, Zhang D, Bai L, Yao R, Yu J (2017). Quercetin decreases insulin resistance in a polycystic ovary syndrome rat model by improving inflammatory microenvironment. Reprod Sci.

[31] Jahan S, Abid A, Khalid S, Afsar T, Shaheen G, Almajwal A (2018). Therapeutic potentials of Quercetin in management of polycystic ovarian syndrome using Letrozole induced rat model: a histological and a biochemical study. J Ovarian Res.

[32] Neisy A, Zal F, Seghatoleslam A, Alaee S (2019). Amelioration by quercetin of insulin resistance and uterine GLUT4 and ERα gene expression in rats with polycystic ovary syndrome (PCOS). Reprod Fertil Dev.

[33] Shah KN, Patel SS (2016). Phosphatidylinositide 3-kinase inhibition: a new potential target for the treatment of polycystic ovarian syndrome. Pharm Biol.

[34] Hong Y, Yin Y, Tan Y, Hong K, Jiang F, Wang Y (2018). Effect of quercetin on biochemical parameters in letrozoleinduced polycystic ovary syndrome in rats. Trop J Pharm Res.

[35] Rezvan N, Moini A, Janani L, Mohammad K, Saedisomeolia A, Nourbakhsh M (2017). Effects of quercetin on adiponectin-mediated insulin sensitivity in polycystic ovary syndrome: a randomized placebo-controlled double-blind clinical trial. Horm Metab Res.

[36] Khorshidi M, Moini A, Alipoor E, Rezvan N, Gorgani-Firuzjaee S, Yaseri M (2018). The effects of quercetin supplementation on metabolic and hormonal parameters as well as plasma concentration and gene expression of resistin in overweight or obese women with polycystic ovary syndrome. Phyther Res.

[37] Rezvan N, Moini A, Gorgani-Firuzjaee S, Hosseinzadeh-Attar MJ (2018). Oral quercetin supplementation enhances adiponectin receptor transcript expression in polycystic ovary syndrome patients: a randomized placebo-controlled double-blind clinical trial. Cell J.

[38] Lu M, Tang Q, Olefsky JM, Mellon PL, Webster NJG (2008). Adiponectin activates adenosine monophosphate-activated protein kinase and decreases luteinizing hormone secretion in LβT2 gonadotropes. Mol Endocrinol.

[39] Kafali H, Iriadam M, Ozardalı I, Demir N (2004). Letrozole-induced polycystic ovaries in the rat: a new model for cystic ovarian disease. Arch Med Res.

[40] Wang F, Yu B, Yang W, Liu J, Lu J, Xia X (2012). Polycystic ovary syndrome resembling histopathological alterations in ovaries from prenatal androgenized female rats. J Ovarian Res.

[41] Rezvanfar MA, Rezvanfar MA, Ahmadi A, Saadi HAS, Baeeri M, Abdollahi M (2012). Mechanistic links between oxidative/nitrosative stress and tumor necrosis factor alpha in letrozole-induced murine polycystic ovary: biochemical and pathological evidences for beneficial effect of pioglitazone. Hum Exp Toxicol.

[42] Doi SAR, Al-Zaid M, Towers PA, Scott CJ, Al-Shoumer KAS (2005). Irregular cycles and steroid hormones in polycystic ovary syndrome. Hum Reprod.

[43] Fukuda S, Orisaka M, Tajima K, Hattori K, Kotsuji F (2009). Luteinizing hormone-induced Akt phosphorylation and androgen production are modulated by MAP kinase in bovine theca cells. J Ovarian Res.

[44] Singh A, Suragani M, Ehtesham NZ, Krishna A (2015). Localization of resistin and its possible roles in the ovary of a vespertilionid bat, *Scotophilus heathi*. Steroids.

[45] Munir I, Yen H-W, Baruth T, Tarkowski R, Azziz R, Magoffin DA (2005). Resistin stimulation of 17α-hydroxylase activity in ovarian theca cells in vitro: relevance to polycystic ovary syndrome. J Clin Endocrinol Metab.

[46] Rak-Mardyła A, Durak M, Gregoraszczuk EŁ (2013). Effects of resistin on porcine ovarian follicle steroidogenesis in prepubertal animals: an in vitro study. Reprod Biol Endocrinol.

[47] Bremer AA, Miller WL (2008). The serine phosphorylation hypothesis of polycystic ovary syndrome: a unifying mechanism for hyperandrogenemia and insulin resistance. Fertil Steril.

[48] Nestler JE (2000). Insulin resistance and the polycystic ovary syndrome: recent advances. Curr Opin Endocrinol Diabetes Obes.

[49] González F, Rote NS, Minium J, Kirwan JP (2006). Reactive oxygen species-induced oxidative stress in the development of insulin resistance and hyperandrogenism in polycystic ovary syndrome. J Clin Endocrinol Metab.

[50] Franks S, Mason H, Willis D (2000). Follicular dynamics in the polycystic ovary syndrome. Mol Cell Endocrinol.

[51] Aguirre L, Arias N, Teresa Macarulla M, Gracia A, Portillo MP. Beneficial effects of quercetin on obesity and diabetes. Open Nutraceuticals J. 2011;4:189–198.

[52] Pawlak K, Mysliwiec M, Pawlak D (2013). Oxidized low-density lipoprotein (oxLDL) plasma levels and oxLDL to LDL ratio—are they real oxidative stress markers in dialyzed patients?. Life Sci.

[53] Bhaskar S, Shalini V, Helen A (2011). Quercetin regulates oxidized LDL induced inflammatory changes in human PBMCs by modulating the TLR-NF-κB signaling pathway. Immunobiology..

[54] Agarwal A, Gupta S, Sharma RK (2005). Role of oxidative stress in female reproduction. Reprod Biol Endocrinol.

[55] Mohamadin AM, Habib FA, Elahi TF (2010). Serum paraoxonase 1 activity and oxidant/antioxidant status in Saudi women with polycystic ovary syndrome. Pathophysiology..

[56] Kadowaki T, Yamauchi T (2011). Adiponectin receptor signaling: a new layer to the current model. Cell Metab.

[57] Satoh H, Nguyen MTA, Miles PDG, Imamura T, Usui I, Olefsky JM (2004). Adenovirus-mediated chronic “hyper-resistinemia” leads to in vivo insulin resistance in normal rats. J Clin Invest.

[58] Palanivel R, Maida A, Liu Y, Sweeney G (2006). Regulation of insulin signalling, glucose uptake and metabolism in rat skeletal muscle cells upon prolonged exposure to resistin. Diabetologia..

[59] Seo JB, Noh MJ, Yoo EJ, Park SY, Park J, Lee IK (2003). Functional characterization of the human resistin promoter with adipocyte determination-and differentiation-dependent factor 1/sterol regulatory element binding protein 1c and CCAAT enhancer binding protein-α. Mol Endocrinol.

[60] Yang L, Li X, Gao L, Zhang Y, Cai G (2012). Suppressive effects of quercetin-3-O-(6 ″-Feruloyl)-β-D-galactopyranoside on adipogenesis in 3T3-L1 preadipocytes through down-regulation of PPARγ and C/EBPα expression. Phyther Res.

[61] Lowes W, Walker M, Alberti KGMM, Agius L (1998). Hexokinase isoenzymes in normal and cirrhotic human liver: suppression of glucokinase in cirrhosis. Biochim Biophys Acta (BBA)-General Subj.

[62] Uno K, Katagiri H, Yamada T, Ishigaki Y, Ogihara T, Imai J (2006). Neuronal pathway from the liver modulates energy expenditure and systemic insulin sensitivity. Science (80- ).

[63] Liu M-L, Ylitalo K, Salonen R, Salonen JT, Taskinen M-R (2004). Circulating oxidized low-density lipoprotein and its association with carotid intima-media thickness in asymptomatic members of familial combined hyperlipidemia families. Arterioscler Thromb Vasc Biol.

